# Evaluation of Culturally Safe, Trauma-Informed Care Training for Service Providers Working with Aboriginal and Torres Strait Islander Peoples

**DOI:** 10.3390/nursrep16100362

**Published:** 2026-10-13

**Authors:** Olivia Mauerhofer, Kimberley Ann Jones, Tess Bright, Skye Stewart, Rebekah Julian, Helen Lucy Henderson, Shakira Onwuka, Danielle Cameron, Caroline Atkinson, Helen Herrman, Catherine Chamberlain

**Affiliations:** 1 Onemda Aboriginal and Torres Strait Islander Health and Wellbeing, Melbourne School of Population and Global Health, The University of Melbourne, Melbourne, VIC 3010, Australia; livmauerhofer4@gmail.com (O.M.); brightt@unimelb.edu.au (T.B.); skye.stewart@unimelb.edu.au (S.S.); rebekah.julian@unimelb.edu.au (R.J.); helenh@unimelb.edu.au (H.L.H.); shakira.onwuka@unimelb.edu.au (S.O.); danielle.cameron@sydney.edu.au (D.C.); caroline.atkinson@wealli.com.au (C.A.); cacham@unimelb.edu.au (C.C.); 2 We Al-Li Pty Ltd., Goolmangar, NSW 2480, Australia; 3 Centre for Youth Mental Health, The University of Melbourne, Melbourne, VIC 3052, Australia; h.herrman@unimelb.edu.au

**Keywords:** trauma-informed care, culturally responsive, health equity, education, healthcare access, nursing

## Abstract

**Background/objectives**: Hospital settings can be unsafe for Aboriginal and Torres Strait Islander peoples, creating healthcare access barriers and perpetuating cycles of inequity. Healthcare service providers play a critical role in improving health equity. The Healing the Past by Nurturing the Future project (HPNF) delivered a training programme that aimed to improve service providers’ awareness, skills, and confidence in providing culturally safe, trauma-informed care. **Methods**: A mixed-methods evaluation assessed the acceptability and changes in self-reported knowledge, attitudes and practice (KAP) of one-day, face-to-face training sessions delivered at a large tertiary hospital in regional Victoria, Australia, from November 2023 to December 2024. Self-reported knowledge, attitudes, and practices were measured on a five-point Likert scale pre-training, post-training, and three months post-training. Participant perspectives were collected through evaluation forms and analysed using an inductive framework approach. **Results**: A total of 547 service providers participated in one of 38 training sessions. Responses to a KAP survey designed for this study showed positive changes in self-reported knowledge, attitudes and practice immediately after training that remained after three months among the smaller number of people that completed the survey. Participants expressed a need and desire for the training. Many highlighted new learning relating to Aboriginal and Torres Strait Islander culture, Australian history, colonisation, and the neurobiology of trauma. **Conclusions**: Aboriginal co-designed face-to-face culturally safe trauma-informed training is highly acceptable for service providers. The HPNF training was followed by positive changes in service providers’ self-reported knowledge, attitudes, and practices in working in a safe and sensitive manner with Aboriginal and Torres Strait Islander peoples. Such training may contribute to changes in health service providers’ capability to provide culturally responsive and trauma informed care. This may reduce healthcare access barriers and contribute to closing the gap in health and wellbeing outcomes and improve health equity.

## 1. Introduction

Healthcare systems globally have historically marginalised priority populations, contributing to poorer health and wellbeing outcomes [1,2]. In Australia, experiences of colonisation and racism continue to shape how Aboriginal and Torres Strait Islander peoples, the First Peoples of Australia, engage with mainstream healthcare systems. Past policies and practices in healthcare settings, such as the denial of traditional ways of birthing, and the removal of children from their families under the guise of ‘assimilation’ has left deep scars on generations of Aboriginal and Torres Strait Islander peoples, resulting in intergenerational trauma [3,4]. Even in the absence of direct experiences of violence or discrimination, Aboriginal and Torres Strait Islander peoples may have a deep fear and mistrust of mainstream systems and institutions. Healthcare providers, including medical staff, midwives, and nurses are uniquely placed to help improve health equity through building trust and providing culturally safe, respectful and responsive care [5,6,7]. Despite their critical role, many healthcare professionals lack the training and skills to provide care that is trauma-informed and specific to the needs of Aboriginal and Torres Strait Islander peoples. These knowledge and skill gaps can lead to harm through ‘triggering’ trauma-related distress symptoms and reducing access to essential health care.

Given the ongoing influence of colonial structures in healthcare, it is critical that providers have an awareness of the historical, political and social determinants that impact Aboriginal and Torres Strait Islander people’s access to mainstream health services. Victoria’s Yoorrook Justice Commission, a formal truth-telling inquiry into colonial injustices against Aboriginal and Torres Strait Islander people, emphasised this need, recommending that the Victorian Government mandate that all health staff working with pregnant women complete training that is designed and delivered by First Nations peoples, to help address bias and build expertise in working in culturally safe ways [8]. These recommendations align with broader shifts in healthcare towards trauma-informed models of care, which provide an alternative approach to traditional biomedical models by acknowledging the impact of past experiences in shaping a person’s health and wellbeing [9,10,11]. Trauma-informed approaches have been shown to improve outcomes in education [12], justice [13], and healthcare settings, including improving patient access to services [14], satisfaction with care and retention [15], and organisational effectiveness [15,16,17]. In perinatal care, trauma-informed approaches help to build trust between families and hospital staff [18,19,20]. However, despite the principles and application of trauma-informed care being well understood [21,22,23], trauma-informed care for Aboriginal and Torres Strait Islander peoples is not well researched or implemented in policy or practice [24,25,26].

The concept of cultural safety is often confused by the saturation of similar terminology, including cultural awareness, cultural competence, and cultural responsiveness [27,28]. Cultural safety was first conceptualised in Aotearoa New Zealand in 1992, and recognises that ongoing awareness and challenging of personal biases, examination of provider–patient power dynamics and the upholding of patient rights are essential for the delivery of healthcare that is safe and responsive to the needs of Indigenous peoples [29]. Currently, cultural competence and/or awareness training is a mandatory requirement for 16 regulated health professions in Australia; however, its effectiveness is uncertain [30,31]. Some argue that these training programmes encourage reflection on how one’s background and values influence care [32], shifting focus from the culture of the patient towards clinician behaviour and practice and the clinical environment [28]. However, whether this translates to changes in practice remains up for debate. One explanation put forth is that training programmes can be treated as a “tick-box” exercise if they are generic, non-ongoing, unevaluated and not delivered alongside broader structural changes that improve the way care is delivered [31,33]. Tujague and Ryan [31] argue that trauma-informed practice is essential for achieving cultural safety and that training must focus on trauma-informed practice alongside cultural education to adequately recognise the complex interplay between culture, history, and trauma. This need for trauma-informed and culturally safe models of care is recognised in Aotearoa, where dominant models fail to recognise the distinct experiences of Māori peoples [34]. Given that Aboriginal and Torres Strait Islander peoples are also affected by trauma in specific ways, there is a need to embed trauma-informed and culturally responsive models of care into policy to ensure that healthcare is responsive to the needs of Aboriginal and Torres Strait Islander peoples [31,34,35].

The Healing the Past by Nurturing the Future (HPNF) project is an Aboriginal-led participatory action research project, aiming to improve support for Aboriginal and Torres Strait Islander parents during pregnancy, birth, and the first 2000 days of life. The first 2000 days are a critical period that can have impacts across the life course due to the significant cognitive, physical and social–emotional development that occurs during this time [36]. Frequent scheduled contact with health services and service providers during the perinatal and early childhood periods provide a unique life course opportunity to provide support to families. For parents experiencing intergenerational trauma, who may be more vulnerable to trauma-related distress, this window presents an opportunity for recovery and interrupting cycles of intergenerational trauma [37,38]. Through four years of co-design with Aboriginal families, Elders, and perinatal service providers, HPNF developed a framework of strategies to increase and improve trauma awareness, recognition, assessment, and support among parents and service providers. A key component of this initiative is a training programme consisting of introductory (online), intermediate (one-day face-to-face) and advanced (four-day face-to-face) health service provider training aimed at building capacity to deliver culturally responsive, trauma-informed care that is tailored to the unique needs of Aboriginal and Torres Strait Islander families. This paper aims to evaluate the acceptability and impact of the HPNF intermediate one-day, face-to-face training on healthcare service providers’ knowledge, attitudes, and practice of culturally responsive, trauma-informed care. By addressing institutional practices contributing to trauma, the HPNF program seeks to empower healthcare providers to offer respectful, supportive, and culturally responsive, trauma-informed care that improves outcomes for Aboriginal and Torres Strait Islander families.

## 2. Materials and Methods

### 2.1. Study Setting and Context

The training was piloted on the traditional lands of the Gunaikurnai people located in Gippsland, Victoria, Australia, in partnership with a regional hospital. The hospital employs approximately 3000 staff, and supports, on average, 880 births per year [39]. Approximately 50 of these births are Aboriginal and Torres Strait Islander babies [40].

Human Research Ethics approval was obtained from the St Vincent’s Hospital Melbourne Human Research Ethics Committee (HREC 239/22).

### 2.2. Participants and Recruitment

Training was delivered and evaluated from November 2023 to December 2024. Initially intended as a small number of training sessions for maternity staff, the scope and timeline of the training was extended in response to unexpectedly high demand from clinical and non-clinical staff. The training was promoted to all staff and mandated for 754 key personnel in priority departments, including midwifery, neonatal care, paediatrics, emergency, mental health, and some areas of allied health (e.g., social work). Non-mandatory attendees included executives, allied health clinicians (e.g., dietitians, physiotherapists, and allied health assistants), business and financial staff, support services (e.g., people and culture), and education and training personnel. Staff were recruited via internal intranet, emails from managers, and promotional flyers placed throughout staff areas of the hospital. Executive level endorsement and active promotion by department managers were critical in supporting recruitment and addressing engagement challenges. All training participants were provided with a participant information sheet and consent form and all participants provided written informed consent. Training sessions were conducted face-to-face on-site at the hospital, as full-day sessions with a maximum of 30 participants per session.

### 2.3. Training Development and Description

The training was developed over 18 months, guided by Nakata’s Interface theory [41] through interdisciplinary collaboration with Aboriginal and Torres Strait Islander and non-Indigenous researchers and healthcare providers. Training content was developed using existing evidence-based guides on trauma-informed approaches to care, including the HPNF Conceptual Framework [37] and the Substance Abuse and Mental Health Services Administration (SAMHSA, Rockville, MD, USA) Framework for Trauma-informed Care [42] [see Supplementary File S1], as well as input from previous co-design workshops [37,43], feedback from parents and service providers [44], and the expertise of the training development group. Team members contributed expertise in complex and intergenerational trauma, midwifery, neuroscience, implementation science, cultural safety training, public health, mental health, health service management, social work, training development and strengths-based healing approaches. A developmental evaluation approach supported the iterative refinement of the training throughout implementation [45]. Early sessions were valuable opportunities to gather participant feedback, which informed ongoing improvements to content and delivery.

Training was delivered by two facilitators from the University of Melbourne, with the aim of co-facilitation by one Aboriginal or Torres Strait Islander and one non-Indigenous facilitator. Most sessions were led by KAJ, a non-Indigenous researcher, with a background in neuroscience and trauma research who has worked in Aboriginal and Torres Strait Islander public health research for three years. When they were available, KAJ delivered the course alongside SS, who is a Wergaia and Wamba Wamba midwife and researcher, and CC, who is a Palawa Trawlwoolway midwife, researcher, and Professor of Indigenous Health. OM, a non-Indigenous researcher, supported the facilitation and delivery of training sessions when CC and SS were unavailable. Although the facilitators were not local to the area, they had established connections with communities in the region.

The training consists of five modules, detailed further in Supplementary File S2. Briefly, the modules covered:

1. The impacts of colonisation on social and emotional wellbeing and the importance of culturally safe, trauma-informed care when working with Aboriginal and Torres Strait Islander peoples.
2. The neurobiology of trauma: Types of trauma, how trauma exposure affects brain development, the stress response, epigenetics, how and why trauma-related symptoms present and feel the way they do.
3. How can we create safe, trauma-aware services?
4. How can we build trusting, respectful relationships with Aboriginal and Torres Strait Islander peoples?
5. The importance of linking with additional supports, such as Aboriginal Community-Controlled Health Organisations (ACCHOs), and looking after yourself (avoiding vicarious trauma and burnout).

The full-day training was delivered using a multimodal learning approach, including didactic presentation, lived experience stories, narrated animation, and short videos with multiple opportunities for practical content application (e.g., small group discussion, case study analysis, experiential, creative, and reflective activities). A practical activity, the Brain Architecture Game [46], was played throughout the day, using pipe cleaners, straws, and ‘Life Experience’ cards to visually demonstrate how early childhood experiences can impact brain development. Training facilitators highlighted the sensitivity of the workshop content, encouraging self-care throughout the workshop, and invited participants to take breaks as needed. Frequent ‘brain breaks’, short, interactive games or opportunities to move, were incorporated to provide pause from the content.

Resources were developed by the project team to supplement the training, including a 100-page training manual with training content, activities, and suggestions for continued learning. A resource table at each session offered lanyards, clinical reference cards and magnets with trauma-informed care reminders, trauma-informed appointment summary pads, and notepads created as part of the HPNF project to reinforce key concepts and support ongoing use in professional settings [Supplementary File S3].

### 2.4. Data Collection Measures

Participant demographics were collected through training registration forms accessed through a Research Electronic Data Capture (REDCap, version 13.10.4 to 15.7.6, Vanderbilt University, Nashville, TN, USA) link emailed to hospital staff or through a QR code on promotional flyers. To assess the impact of training, Knowledge, Attitudes, and Practice (KAP) survey data were collected immediately pre-, immediately post-, and three months after training. Training Evaluation data were collected once, immediately following completion of the training.

#### 2.4.1. Knowledge, Attitudes, and Practice Survey

Training participants’ knowledge, attitudes, and practices were measured using a 13-item, researcher-developed survey adapted from a previously developed tool [47], specifically to evaluate this training. Items were iteratively developed in alignment with the training curriculum [48]. The KAP survey has not undergone formal psychometric validation, as it was designed as a purpose-built evaluation tool for this training rather than a generalisable measure of trauma-informed care competency more broadly. The knowledge domain of the survey included five items, which assessed knowledge of Closing the Gap targets, local services and supports for Aboriginal and Torres Strait Islander people, the impacts of colonisation and knowledge of trauma-informed approaches. Items within the attitude domain assessed beliefs about Aboriginal and Torres Strait Islander families and participants’ own perceptions of trauma-informed practice. Items within the practice domain assessed behaviours related to collaboration with external services and approaches to providing care to families. Participants not employed in patient-facing roles had the option to select “not relevant to my role” for practice questions related to the provision of patient care. KAP responses were assessed on a five-point Likert scale, where 1 = strongly disagree and 5 = strongly agree [49]. The three-month follow-up KAP survey also contained open-ended questions for participants to provide feedback regarding individual, departmental, and organisational changes relating to trauma-informed care following training. Pre- and post- surveys were collected in paper format on the training day, while the 3 month follow-up survey was administered via an emailed REDCap link (version 13.10.4 to 15.7.6, Vanderbilt University, Nashville, TN, USA) [50,51].

#### 2.4.2. Training Evaluation

The training evaluation form consisted of 11 closed-ended questions using a five-point Likert scale (1 = Strongly disagree and 5 = Strongly agree) to measure perceptions of the training’s quality, clarity, engagement, overall satisfaction, and usefulness of individual modules. Five open-ended questions were asked about participants’ experiences and recommendations regarding the training [Supplementary File S4].

### 2.5. Analysis

Quantitative KAP survey data was entered into REDCap, then exported, cleaned, and analysed using Stata version 18.0 (StataCorp, College Station, TX, USA. Each item was scored on a five-point Likert scale (1 = strongly disagree, 5 = strongly agree) [Supplementary File S5], with higher scores indicating greater awareness of and more positive feelings towards concepts covered in training, and better practice in implementing strategies taught in training. To ensure that a score of 5 consistently represented the most desirable response, negatively worded items were reverse-scored prior to analysis. For each participant, mean item scores within each domain were calculated, with missing responses excluded from this calculation on a per-participant basis. Mean domain level scores were then calculated for knowledge and attitudes pre-, and post-training, and at three-month follow-up. Mean practice scores were only reported pre-training and at 3 month follow-up, as immediate post-training scores cannot reflect a true change in practice. As no identifier codes were employed during data collection, participant responses were unable to be matched across the three collection time points. Although most participants completed both surveys, independent sample t-tests were calculated to compare baseline scores with post-training and three-month follow-up scores. This approach violates the assumption of independence, the impact of which is later discussed in limitations. Descriptive statistics were calculated for responses to the training evaluation form. Qualitative data was stored in both NVivo version 13 (QSR International, Melbourne, Australia) and Microsoft Excel (Microsoft 365, Microsoft Corporation, Redmond, WA, USA).

The training team (OM, KAJ, CC, SS, SO, HLH) met within 24 h of each training session to review training evaluation forms, allowing for reflexive discussions about the training content and delivery. These team discussions provided the opportunity to collaboratively develop a coding framework for the data and generate early themes. An inductive framework analysis [52] was used to organise and interpret the data and to further develop themes. OM coded the open-ended questions, consulting with other team members on the coding process and theme development, to ensure a high degree of agreement about interpretation of the data.

## 3. Results

Five hundred and forty-seven hospital staff attended 38 training sessions. Professional discipline and years worked in role and at the organisation are described in Table 1. Almost three-quarters of attendees were clinical staff (74.2%, *n* = 406), 24.0% were non-clinical staff (*n* = 131), and 1.8% did not disclose their role (*n* = 10). Sixty percent of the staff from mandatory department areas attended a training session (*n* = 458).

The most frequent avenue of training recruitment was through a manager (32.70%, *n* = 152), followed by recommendation from colleagues (25.6%, *n* = 119), posters (20.4%, *n* = 95), and the staff newsletter (9.7%, *n* = 45). Just over ten percent (11.0%, *n* = 51) of participants heard about the training through ‘other’ avenues.

### 3.1. Impact on Knowledge, Attitudes, and Practice

Of the 547 healthcare service providers who attended a training session, 94.52% completed a pre-training KAP survey (*n* = 517), 94.33% completed a post-training survey (*n* = 516), and 35.59% (*n* = 184) completed a three-month follow-up survey.

**Figure 1:**
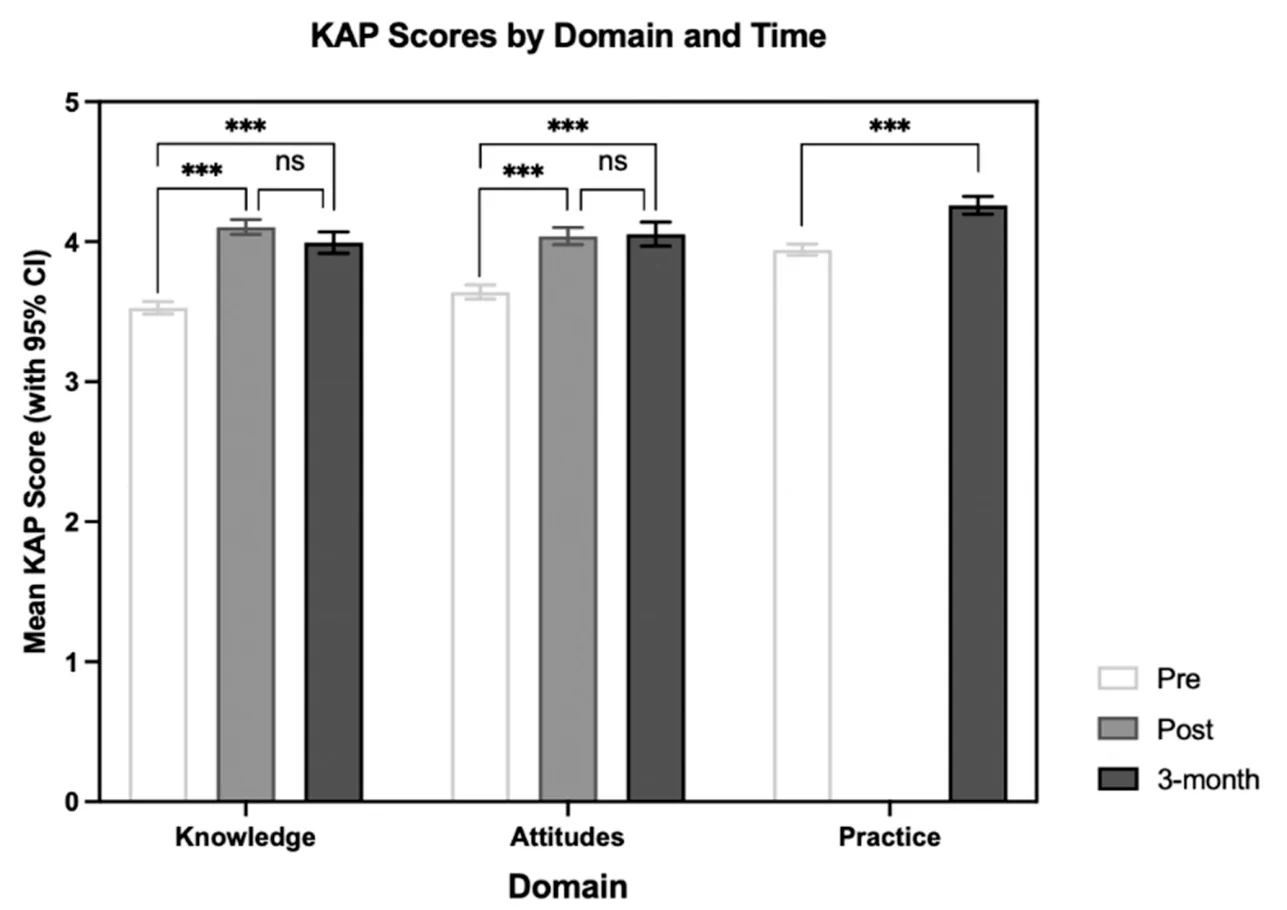
Mean scores with 95% confidence intervals (CI) for knowledge, attitude and practice (KAP) domains pre-training, post-training and 3 month follow-up (*** *p* < 0.001, ns = not significant).

A significant increase in self-reported knowledge scores was observed post-training, with mean (SD) scores rising from 3.53 (0.52), 95%CI [3.48–3.57] pre-training, to 4.11 (0.62), 95% CI [4.05–4.15], *p* < 0.001 post-training. Although knowledge scores declined slightly at three-month follow-up (3.99 (0.53), 95% CI [3.91–4.07], *p* = 0.03), they remained significantly higher than baseline levels (*p* < 0.001). Self-reported attitude scores significantly increased from 3.64 (0.59), 95% CI [3.59–3.69] pre-training to 4.04 (0.70), 95% CI [3.98–4.10], *p* < 0.001 post-training. Scores remained higher at three-month follow-up than at baseline amongst those that responded to the survey (4.05, (0.60), 95% CI [3.97–4.14], *p* < 0.001). A significant increase in self-reported practice scores was also observed, with mean scores rising from 3.94 (0.47), 95% CI [3.90–3.98] pre-training to 4.26 (0.44), 95% CI [4.19–4.33], *p* < 0.001, at 3 month follow-up. The mean scores in each domain at each timepoint are displayed in Figure 1.

### 3.2. Acceptability of Training

The training evaluation was completed by 85% of participants (*n* = 465). Satisfaction levels were high across all domains, with between 96.97% and 98.70% of participants agreeing or strongly agreeing to all questions (Table 2). All five modules received high scores for perceived usefulness (Table 3).

Five hundred and thirty-eight participants responded to the open-ended questions, resulting in 1765 comments. Three themes were generated from the responses: (1) service providers value and want the training; (2) the training addresses important knowledge gaps; and (3) the training format supports participant engagement and collaboration.

#### 3.2.1. Service Providers Value and Want the Training

**“*Should be mandatory in all hospitals in Australia*”: The training content is relevant and useful.**

Participants consistently expressed appreciation for the training, with many suggesting that the training should be compulsory for all healthcare providers “*to improve cultural competence*”. Many commented on the relevance and practical benefit that the training provided in their area of work.

*“Thank you. I had low expectations for today’s training but it was brilliant and I’m glad that I attended.”*

The real-world application was a strength. Participants valued the practical examples, tips and strategies as key learning and enjoyment. Resources, including the HPNF appointment summary pad and training manual, were reported to be useful in practice three months following training.

*“[I learnt new] practical ways of engaging families/building trust”*

*“I have handwritten appointments using the note pad we were given.”*

*“The appointment summary booklet helped [a] lot.”*

Participants described applying the training “*to my everyday activities in small ways*”, including increased awareness of culturally responsive communication strategies, adjusting language, and adopting a yarning style:

*“I feel I’ve engaged in a more relaxed and low-pressure way towards my First Nations patients. This includes more flexibility in how they engage with our healthcare service”*

**“*Today was so valuable to me*”: Participants find the training content meaningful.**

As well as finding the content useful and relevant, the training was described as highly engaging and meaningful. Many participants expressed appreciation for content regarding cultural practices and items of significance. Learning about items such as the possum skin cloak and coolamon resonated deeply with participants:

*“Dadirri—what a beautiful concept.”*

*“[I liked] some of the discussion about traditional practices/cultural activities”*

*“possum blankets < 3”*

Additional topics such as trauma physiology, communication strategies, social–emotional wellbeing, history of the local area, and available services were highlighted as valuable and meaningful:

*“I have learned about more appropriate ways to communicate with [Aboriginal and Torres Strait Islander] people. I’ve also gained knowledge of resources available to help my knowledge as well as patients”*

#### 3.2.2. The Training Addresses Important Knowledge Gaps

Participants identified gaps in their understanding, highlighting the need for continued education to improve culturally responsive, trauma-informed practice. Responses to the open-ended question “Did you learn anything new?” highlighted that *“most of the content was all new”* to many participants, with one participant noting*, “all of it [the content] was new”.*

*“This box isn’t big enough to write all I have learnt.”*

Some participants expressed that, while they did not learn anything new, the training provided a *“very good refresher on the topic”*. It also provided participants with an opportunity to consolidate previous education and training.

**“*I need more education*”: Service providers need more cultural education.**

The cultural aspects of the training were an identified knowledge gap. Many participants reported new learning relating to Aboriginal and Torres Strait Islander culture, social and emotional wellbeing, colonisation, and Australian history.

*“[I learnt about] the rich culture and spirit of the Indigenous people prior to colonisation”*

*“Learned about the trauma that First Nations have endured. This was not addressed in my schooling and has been eye opening. The generational trauma is very interesting and scary”*

Additionally, participants reflected on the need to increase cultural education. One participant reflected that they *“need more [cultural] education”*, while others reflected that they would like *“to learn a little bit more about our Gunaikurnai land”.*

*“[I learnt about] the history of the Gunaikurnai land and about support networks locally that I didn’t know about.”*

**“*Really affirming to explain trauma…It’s more palatable*”: Service providers lack awareness of the neurobiological basis of trauma.**

The physiology and neurobiology of trauma were a key component of the training. Insights into intergenerational trauma, stress responses and the impact of trauma on brain development were highlighted as valuable new learning. Numerous participants identified concepts related to epigenetics as novel and highly valuable aspects of the training. Some participants reported that the explanation of trauma through neural mechanisms was both simple and impactful.

*“Hearing about epigenetics and the physiology behind trauma (for my practical brain) made a lot of sense”*

*“[I learnt] how trauma can impact brain development and how trauma can affect a whole culture for generations”*

**“*I didn’t know the hospital had an [Aboriginal Liaison Officer] ALO*”: Participants have limited awareness of Aboriginal-specific services.**

Many participants highlighted increased knowledge *“about local services, the role of ALOs and how valuable they are”,* with many reporting that the training was valuable for meeting the Aboriginal Hospital Liaison Officers (AHLOs), connecting with other service providers, and learning about external health services.

*“New resources, met new people to resource. Consolidated a lot of knowledge.”*

*“[I learnt about] how to contact the hospital ALO”*

At three-month follow-up, participants reported increased confidence and greater engagement with AHLOs and Aboriginal Community Controlled Health Organisations (ACCHOs), stemming from their newly increased awareness of barriers to healthcare access:

*“I have been more mindful of what we learnt in the training when I am caring for an Aboriginal or Torres Strait Islander consumer on my ward, and ensure support is offered through our ALO”*

*“More proactive in looking at ways to close the gap-more, engaged with AHLO and community services in improving health outcomes. Regular meetings with [local ACCHO].”*

#### 3.2.3. The Training Format Supports Engagement and Collaboration

**“*The facilitation was expert*”: Dynamic and enthusiastic facilitation fosters engagement.**

Qualitative evaluations consistently highlighted the engaging, knowledgeable, and passionate facilitation as a crucial factor contributing to engagement. Numerous participants specifically mentioned or expressed gratitude toward the facilitators for creating an open and supportive environment to address complex topics and questions perceived as challenging for health professionals:

*“[Facilitator 1] and [facilitator 2] were great presenters and demonstrated a really strong understanding and passion for what they were talking about which helped make it more engaging”.*

*“Both [facilitator 1] and [facilitator 2] have bubbly personalities and were very engaging”.*

**“*Interactive, but not in a way that makes you feel uncomfortable*”: Multimodal delivery supports different learning styles.**

Participants valued the interactive and multimodal delivery format, with many highlighting how the training session had a *“good mix of learning [and] breaks” which “kept things interesting and engaging”*. Participants highlighted the inclusion of the Brain Architecture Game [45] as particularly impactful and a key factor in their overall enjoyment of the training:

*“Loved how interactive the whole day was, such practical application to midwifery and life”*

*“I usually loathe the groupwork activities, but these were really different and interesting, very enjoyable to participate in. Stories and lived experience materials of Aboriginal peoples (*e.g.*, videos).”*

*“The straw “building the brain” was a wonderful way to visually understand how trauma + supports can make a difference to someone’s life journey. Thank you < 3”*

Many participants described connecting with individuals across different specialisations, units, and departments as highly beneficial. While some participants noted that *“listening to other participants perspectives was fascinating”,* and that *“being able to learn from others of different disciplines”* enriched the training, some participants felt the training would benefit from a broadened scope:

*“Would be useful if this was perhaps a little less focused on maternity, otherwise a fabulous training day that should be attended by all hospital staff.”*

*“A really enjoyable and informative day”: Trauma-informed delivery improves experience.*

Additional qualitative responses revealed that the *“extra touches”,* such as music, lived experience stories, the training manual and resources for participants to keep, the catering, and having lunch outside on yarning mats made the day much more enjoyable.

*“Great job guys! great venue/room, I loved the frequent breaks, food was delish, loved eating lunch outside, excellent presenting from your team, and the training activity book looks like a really good resource. thanks.”*

*“It was fantastic having the brain breaks to break up a heavy subject. Having the music throughout the day created a beautiful vibe.”*

Many participants highlighted that while sessions similar to the HPNF training are often daunting and can be challenging to attend, the trauma-informed delivery style and content “*created a safe place*” for all:

*“Thank you for this. I cancelled 3 times before coming as I was worried, not sure what of because I am Māori with a similar history, it can be triggering but this was well put together. I hope everyone in Australia can do this training more regularly. Thank you for your kindness and compassion.”*

*“Very engaging and informative. I loved the option of colouring throughout—it helped my focus—I hope it didn’t come across rude. As someone with complex PTSD I dread sessions around trauma informed care—but this was wonderful.”*

Few responses highlighted barriers to engagement and satisfaction with the training. While most participants engaged reflectively with the training content, noting that the training *“presented a very heavy, hard topic with sensitivity and without getting bogged down”*, two comments highlighted discomfort with one activity*: “[the privilege walk activity] can be pretty humiliating or [suggest] people are white privileged”.* Some other comments made suggestions for improvement, suggesting that the length and volume of content would be better suited to being disseminated over two shorter days, rather than a single full day, to help accommodate participants’ energy levels.

*“Probably went for too long—1/2 day maybe enough, and specified to certain job roles”*

*“I feel we may have missed time on activities due to the pressure of so much info to deliver”*

## 4. Discussion

The HPNF culturally responsive, trauma-informed care training evaluation demonstrated increases in healthcare providers’ self-reported knowledge, attitudes, and practice. These changes were sustained among the smaller number of people who completed the 3 month follow-up survey. Positive qualitative reflections suggested that education and training programmes can meaningfully support the delivery of safer care.

This study adds to the growing body of evidence supporting the importance of trauma-informed care training and cultural safety education for healthcare providers. Although cultural education programmes have been criticised as superficial, research suggests that they can meaningfully enhance understanding among healthcare providers [53,54,55,56]. An evaluation of a workshop aimed at improving culturally responsive cancer care found significant improvements in nine of fourteen indicators relating to confidence in providing culturally sensitive care, with eight sustained two months later [54]. However, some studies report limited or no sustained gains following training, such as a cultural education programme for 44 Emergency Department staff, which changed perceptions, but not attitudes [53]. Similarly, an evaluation of a training program delivered to 122 health professionals and students found that while training increased positive attitudes, no significant differences were found at two-month follow up [57]. Unlike these studies, a key strength of our study was the large sample size. Similar large-scale studies, such as a quantitative evaluation of cultural awareness training for over 600 healthcare providers by Kerrigan and colleagues [55] reinforce the significance of our findings; however, our use of a mixed methods design adds further depth by combining statistical significance with rich qualitative feedback.

The literature similarly supports the value of trauma-informed care training. A review of 24 trauma-informed care training studies by Purtle and colleagues [58] found that 17 of the studies using a pre-test/post-test design reported significant post-training improvements in staff knowledge, attitudes, and behaviours, with seven maintaining improvements beyond one month. In the perinatal context, Long and colleagues [59] found significant improvements in attitudes towards trauma-informed care, where midwives who had participated in an education workshop reported significantly higher scores for positive attitudes compared to those who had not, which were sustained at 6 months. Despite training interventions appearing to be effective, Purtle and colleagues advise caution when interpreting these results, suggesting that the strength of evidence for the effectiveness of these programmes is hindered by inadequate and heterogeneous evaluation methods, such as the use of single group pre-test/post-test designs, with short follow-up periods, and inconsistent assessment instruments, which limit the ability to determine the impact of training programmes at an organisational level [30,58].

While trauma-informed care and cultural responsiveness training are often delivered separately, the integration of both was a key strength of the current study. Many participants highlighted the truth telling and cultural content as the components of the training they liked most, despite the HPNF training being framed and delivered as trauma-informed care training with a cultural lens, rather than cultural responsiveness training. Some noted that these concepts had been absent in previous higher education training, reflecting broader gaps in Australia’s education system, where Aboriginal and Torres Strait Islander cultures, histories, and knowledges were excluded from classroom curricula until 2010 [60]. Additionally, given that the Australian heath workforce predominantly comprises non-Indigenous people, many of whom were born or trained overseas [61], it is unsurprising that health professionals may have limited awareness of Aboriginal and Torres Strait Islander cultures or Australian history. This context may explain participants’ keen interest in learning more about local Aboriginal culture, and appreciation for the lived experience stories shared by Aboriginal facilitators. These findings align with similar evaluations of large-scale training programmes, such as the cultural awareness program by Rissel and colleagues [56], that found cultural awareness training to be overwhelmingly positive and relevant for health service providers, with participants gaining new insights into colonisation, culture, and kinship systems. Kerrigan and colleagues [55] similarly found significant participant enjoyment of cultural content, highlighting the value and need for ongoing, locally relevant cultural education. In the current study, facilitators noted challenges in delivering local content comprehensively due to the need for cultural expertise from traditional knowledge holders, and the restrictions on scope for the purposes of the project. Ongoing involvement and support from the AHLOs throughout the training delivery was critical to ensure the training remained culturally appropriate.

The neurobiological content was another particularly engaging component for service providers, potentially contributing to the training’s success. The scientific explanation of key concepts relating to the stress response, the impact of trauma on brain development, and epigenetics can help demystify behaviours that are often misunderstood or misjudged [62]. By understanding that trauma affects brain structure and function [63], and that certain behaviours stem from automatic, unconscious responses to stress [64], participants may have been able to expand their capacity to empathise with how individuals respond to stress and trauma. This is reinforced by studies that have shown the effectiveness of neuroeducation, which offers accessible, compelling, and relatable insights into brain function, behaviour, and learning patterns [65,66]. Neuroeducation is frequently used in educational contexts [66,67], and has more recently been adopted in healthcare settings to help clinicians conceptualise trauma, guide assessment, implement interventions and communicate with clients and other clinicians [65]. As such, integrating neurobiological content into training may help to deepen healthcare providers’ understanding of trauma and help clinicians respond more effectively to people during episodes of care.

Training evaluations highlighted several effective strategies for participant engagement and satisfaction. While full-day trainings are often criticised for their long duration and information load [55,56], participants reported that the use of varying presentation styles, brain breaks, interactive activities and the provision of hands-on activities like colouring sheets helped reduce fatigue. These approaches, combined with facilitator enthusiasm and expertise, align with research showing that enthusiastic delivery and focus aids support learning motivation and knowledge application [68,69,70,71,72]. Iterative improvements to training content and delivery based on participant feedback, such as the inclusion of the Brain Architecture Game [46] to increase practical application and content relating to Clinical Yarning [73] to improve flow and structure in Module 4, were critical for maintaining engagement. Programme delivery from an external organisation may have further increased engagement due to variety of formats and deliveries compared to standard in-house training [74]. Importantly, participants reported feeling safe throughout the training, supporting the feasibility of an interactive and trauma-informed delivery.

There are several limitations that should be taken into consideration when interpreting this study’s findings. Firstly, the KAP survey was adapted from a previous study [47], and was not psychometrically validated beyond face validity. Whilst validation was beyond the scope of this study, the possibility of measurement bias should be acknowledged. Additionally, the use of a self-report survey may have introduced social desirability bias, potentially skewing results [75,76]. The pre–post-survey design also introduces the risk of response shift bias, where participants’ evolving understanding of concepts may alter how they interpret and respond to questions post-training. Additionally, considerably fewer participants responded to the 3-month follow-up survey (35.59%, *n* = 184). This low response rate may have introduced selection bias, whereby those that responded to the 3-month follow up survey may differ systematically from those who did not [77]. This may have under- or over-estimated changes in self-reported knowledge, attitudes and practice over time. Due to the unpaired and anonymous design of this study, we were unable to conduct analyses to investigate potential differences in participant characteristics; as such, conclusions about the maintenance of improved scores 3 months post-training should be made with a degree of caution. Future studies should aim to investigate what characteristics, if any, lead to a higher likelihood of sustained engagement and higher-scoring responses at different time-points. This information may enable the development of specialised training programmes and initiatives that have sustained impact over time. As previously stated, the assumption of independence has been violated in our analysis of the observed changes in pre–post mean KAP scores. In this instance, this violation has occurred due to repeated construct measurement within the same sample, which typically results in positive correlations between measurements. Under this condition, independent sample t-tests have been demonstrated to provide a conservative estimate of significance, meaning that it is unlikely that the violation has resulted in a significant result where none exists [78]. Regardless, future studies should aim to ensure that a paired design is employed to match responses across time points to address this limitation. Importantly, the current study did not assess impact on patient experience outcomes; as such, we are unable to determine whether changes in self-reported practice have translated to changes in practice and care delivery. This presents an important area for future research that may contribute to improvements in training design and delivery, and broader structural changes that contribute to sustained practice changes. The absence of a control group was a further limitation of this study. Given that the training aimed to be delivered as widely as possible in this implementation study across staff, recruiting a comparable untrained sample for comparison was not feasible. Consequently, changes in self-reported KAP following training cannot be causally attributed to the training itself, and the possibility of other factors, such as concurrent workplace initiatives or repeated exposure to the survey, contributing to the observed changes cannot be ruled out.

While the training was intended to be co-facilitated by an Aboriginal and Torres Strait Islander facilitator, this was not always feasible. Across all 38 sessions, 16 sessions (42%) were delivered with an Aboriginal co-facilitator, with most of these occurring in the first 17 sessions held before August 2024. Due to high demand, we added a final tranche of 11 sessions. For these final sessions, no Aboriginal co-facilitators were available. However, to mitigate this, facilitators centred Aboriginal and Torres Strait Islander voices, stories, and lived experiences through pre-recorded videos, case studies and anonymised patient interview data captured during the pre-implementation phase of the HPNF project. Session facilitators took care to openly acknowledge their own non-Indigenous positionality to promote allyship and help lighten the cultural load [79,80]. Nevertheless, the presence or absence of Aboriginal cofacilitators may have influenced participants’ experiences of the training in ways that have not been examined in this study. Finally, this study was conducted at one hospital; as such, our findings are only applicable to this specific context and may not be generalisable across other health service settings due to differing social, cultural, historical and practice contexts. Additional research is being conducted to evaluate the training in other settings.

## 5. Conclusions

Barriers to healthcare for Indigenous peoples, including Aboriginal and Torres Strait Islander people, are closely linked to trauma and culturally unsafe care within healthcare services [81]. Strengthening service provider awareness of cultural safety, the importance of trauma-informed and culturally responsive care and how this can be provided may lead to changes in practice and care delivery that help reduce access barriers and improve care quality for Aboriginal and Torres Strait Islander peoples [31]. The HPNF training programme evaluation suggests improvements in self-reported knowledge, attitudes and practice scores among respondents. Embedding ongoing locally tailored training programmes that combine trauma-informed practice with cultural education within health systems may improve service providers’ capability to provide culturally responsive and trauma-informed care. This improved capability may improve patient experiences and access, making this a potentially effective strategy to improve health equity for Aboriginal and Torres Strait Islander peoples in Australia. Future implementation initiatives should examine how staff education can be embedded within broader structural approaches to improving care and investigate the conditions required to ensure sustainability over time.

## Figures and Tables

**Table 1:** Characteristics of participants who attended the training.

Participant Characteristics	*n* (%)
Role in hospital (*n* = 547)	
Nurse	166 (30.35)
Allied Health ^1^	87 (17.37)
Mental Health	63 (11.52)
Nurse Midwife/Midwife	43 (7.86)
Business, Financial and Hospital Services	40 (7.32)
Executive	29 (5.30)
Research	24 (4.39)
Education and Training	20 (3.66)
Patient Support Services ^2^	20 (3.66)
Doctor (Medical Staff)	15 (2.74)
Aboriginal Health	11 (2.01)
Public Health	8 (1.46)
Years in Current Role (*n* = 487)	
0–≤5 years	309 (63.45)
>5–≤10 years	71 (14.58)
>10 years	107 (21.97)
Years in Organisation (*n* = 487)	
0–≤5 years	300 (61.60)
>5–≤10 years	71 (14.58)
>10 years	116 (23.82)
Recruitment Channel (*n* = 465)	
Manager	152 (32.70)
Colleagues	119 (25.60)
Poster	95 (20.43)
Newsletter	45 (9.68)
Other	51 (10.97)

^1^ Including social work. ^2^ Roles that are patient-facing and non-clinical.

**Table 2:** Results of training evaluation.

Item	Agree/Strongly Agree
The training was engaging (*n* = 465)	98.06%
The training was easy to understand (*n* = 464)	98.70%
There were enough practical activities (*n* = 461)	97.61%
The training materials provided were useful (*n* = 465)	98.28%
The training was high quality (*n* = 463)	98.27%
I would recommend this training (*n* = 462)	96.97%

**Table 3:** Results of the usefulness of each module.

Item (“The Following Sessions Were Useful for Me”)	Mean (SD)
Module 1 (*n* = 462)	4.64 (0.61)
Module 2 (*n* = 462)	4.65 (0.64)
Module 3 (*n* = 461)	4.61 (0.62)
Module 4 (*n* = 459)	4.63 (0.64)
Module 5 (*n* = 454)	4.59 (0.65)

## Data Availability

The qualitative data generated and analysed during this study are not publicly available due to ethical and privacy constraints. De-identified quotes from the datasets are included in the paper. Quantitative data generated and analysed during this study are available from the corresponding author upon reasonable request.

## Supplementary Materials

The following supporting information can be downloaded at https://www.mdpi.com/article/10.3390/nursrep16100362/s1. File S1: HPNF eight core values and Substance Abuse and Mental Health Servies Administration (SAMHSA) assumptions and principles [37,42]; File S2: Detailed overview of the HPNF culturally safe, trauma-informed care training learning aims and outcomes; File S3: Images of HPNF culturally safe and trauma-informed resources for service providers; File S4: Evaluation form for the HPNF training; File S5: Knowledge, Attitudes, and Practice survey items; File S6: The TIDieR (Template for Intervention Description and Replication) Checklist.

## Public Involvement Statement

There was no public involvement in any aspect of this research.

## Guidelines and Standards Statement

The training intervention has been described in accordance with the Template for Intervention Description and Replication (TIDieR) Checklist. No other formal reporting guidelines were used in the preparation of this manuscript.

## Abbreviations

The following abbreviations are used in this manuscript:

ACCHO	Aboriginal Community Controlled Health Organisation
AHLOs	Aboriginal Hospital Liaison Officers
HPNF	Healing the Past by Nurturing the Future
KAP survey	Knowledge, Attitudes, and Practice Survey
