# Supplementary material for: Evaluation of Culturally Safe, Trauma-Informed Care Training for Service Providers Working with Aboriginal and Torres Strait Islander Peoples
Source: Nurs Rep. 2026 Oct 13;16(10):362. doi: 10.3390/nursrep16100362 (PMC13708140; doi:10.3390/nursrep16100362)
Supplement: Supplementary file 1 [file nursrep-16-00362-s001.zip › nursrep-4496261-supplementary.pdf]

Supplementary materials

Supplementary File S1

Title of data: HPNF eight core values and Substance Abuse and Mental Health Servies Administration (SAMHSA) assumptions and principles.
Description of data: Description of the core values guiding the development of the HPNF intermediate training. It defines the eight core HPNF values, codesigned with Aboriginal and Torres Strait Islander community members, and the assumptions of the Substance Abuse Mental Health Services Administration Framework for trauma-informed care.

Healing the Past by Nurturing the Future Core Principles [1]

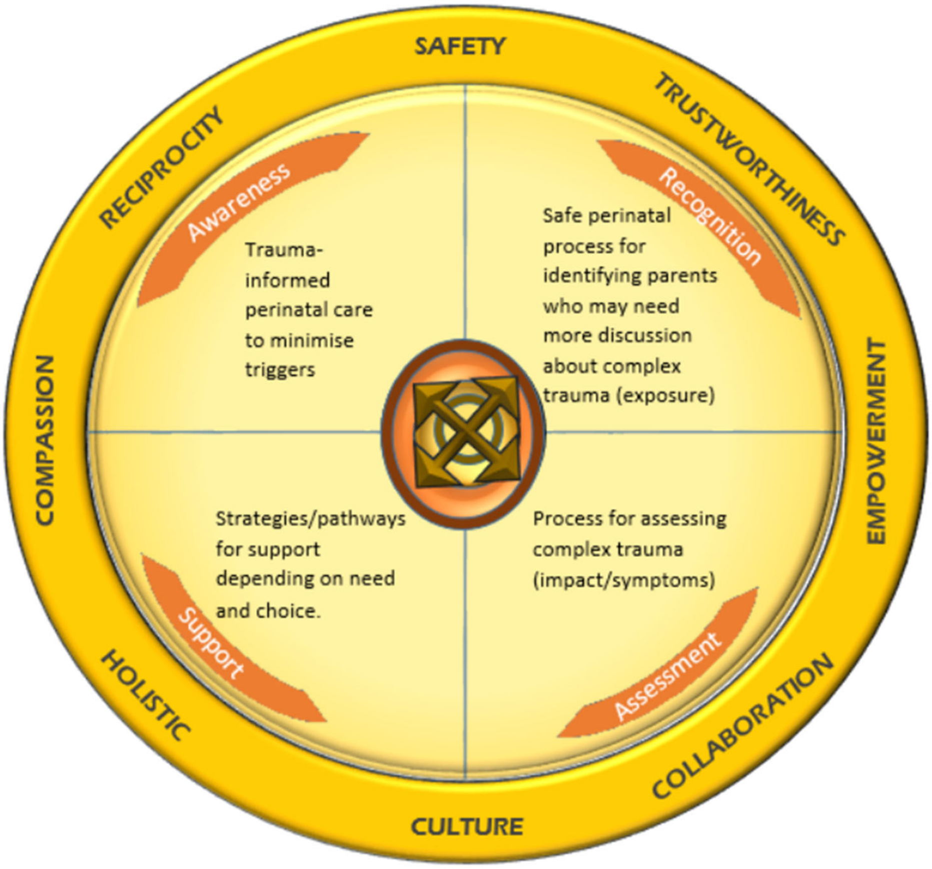

Substance Abuse Mental Health Services Administration (SAMHSA) Framework for trauma-informed care [2]

1	Safety	Safety in physical settings and interpersonal interactions
2	Trustworthiness and Transparency	Operations are conducted and decisions are made with transparency, consistency, respect, and fairness so as to build and maintain trust.

3	Peer Support	Support from those with lived experiences of trauma, or in the case of children with history of trauma, their family members
4	Collaboration and Mutuality	Partnering, levelling of power differences between and among staff and clients
5	Empowerment	Individuals' strengths and experiences are recognised and built upon
6	Cultural, Historical and Gender Issues	Organisation moves beyond the cultural stereotypes and biases.

Supplementary File S2

Title of data: Detailed overview of the HPNF culturally safe, trauma-informed care training learning aims and outcomes.
Description of data: Overview of the HPNF intermediate training titles, aims, and success criteria for each module.

CULTURALLY SAFE TRAUMA-INFORMED CARE TRAINING

Module Overview	By the end of the module, participants will be able to:
**Module One: The impact of colonisation on Aboriginal and Torres Strait Islander social and emotional wellbeing**	
Understand Aboriginal and Torres Strait Islander social and emotional wellbeing (SEWB) and the impact of colonisation on health equity. Appreciate the importance of trauma-aware healing-informed care.	Describe the seven domains of wellbeing that characterise Aboriginal and Torres Strait Islander definitions of SEWB.
	Explain the impacts of colonisation on Aboriginal and Torres Strait Islander SEWB, trust in health services and health equity.
	Describe the progress of the Closing the Gap targets and reasons underpinning this.
	Discuss windows of opportunity for healing trauma.
**Module Two: Physiology and neurobiology of trauma**	
Understand the types of trauma, basic overview of the neurobiology of trauma, and how these impact behaviour later in life.	Identify causes and symptoms of different types of trauma (PTSD, Complex PTSD, intergenerational trauma).
	Explain the stress response and how it can become dysregulated after trauma exposure.
	Explain the changes to brain structure and function that can occur when children are exposed to trauma.
	Explain how epigenetic changes may facilitate the inheritance of trauma-related responses across generations.
	Describe how challenging behaviours and presentations may be explained by previous trauma and should be viewed as understandable, adaptive coping strategies.
**Module Three: How can we create safe, trauma-aware services?**	
Consider what trauma-aware healing-informed care looks like, and reflect on how to create physically, emotionally, and culturally safe spaces.	Describe and apply the concept of universal precautions in relation to trauma-informed care.
	Identify elements of physical environments that help people feel welcome and safe.
	Describe and apply strategies to create emotional safety.
	Explain basic strategies to create cultural safety and the importance of cultural safety training to further this knowledge.
**Module Four: How can we build trusting, respectful relationships with Aboriginal and Torres Strait Islander people?**	
Develop strategies for building trusting relationships with Aboriginal and Torres Strait Islander people.	Understand and apply thoughtful communication strategies including clinical yarning, deep listening.
	Describe the difference between indirect and direct language. Apply invitational communication techniques.
	Understand and apply strategies to empower through providing choice.
	Understand and apply strengths-based approaches.
**Module Five: The importance of linking to further support and looking after yourself**	
Understand what supports Aboriginal and Torres Strait Islander people may want and need, link to Aboriginal Community Controlled Organisations. Minimise service provider vicarious trauma and burnout.	Describe what kinds of supports Aboriginal and Torres Strait Islander families may want and need.
	Recognise the need for collaboration, authentic partnerships, and support to access services.
	Explain vicarious trauma and strategies to minimise stress and burnout and promote wellbeing at work.

Supplementary File S3

Title of data: Images of HPNF culturally safe and trauma-informed resources for service providers.
Description of data: Image of the HPNF merchandise available for service providers attending the training. These include a participant workbook, lanyards, clinical reference cards and magnets with trauma-informed care reminders, trauma-informed appointment summary pads, and notepads.

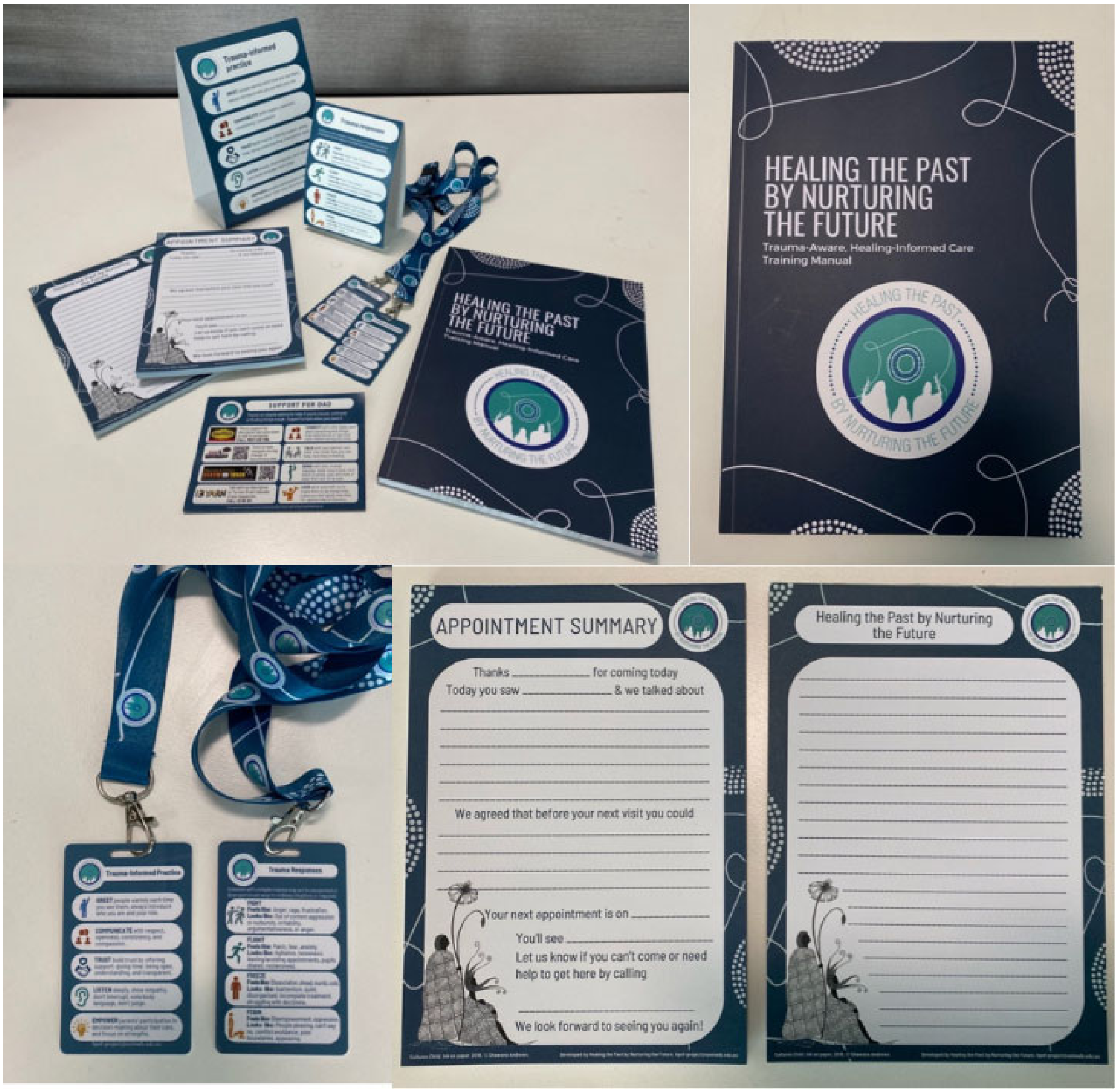

Supplementary File S4

Title of data: Evaluation form for the HPNF training.
Description of data: An evaluation form given to workshop participants at the end of each training session to measure perceptions of the training's quality, usefulness, learnings, key areas of enjoyment, and recommendations regarding the training.

HPNF TRAINING EVALUATION

HEALING THE PAST BY NURTURING THE FUTURE

Date:

Your role:

Please rate how much you agree with the following statements

	Strongly disagree	Disagree	Neutral	Agree	Strongly agree
The training was engaging	☐	☐	☐	☐	☐
The training was easy to understand	☐	☐	☐	☐	☐
There were enough practical activities	☐	☐	☐	☐	☐
The training materials provided were useful	☐	☐	☐	☐	☐
The training was high quality	☐	☐	☐	☐	☐
I would recommend this training	☐	☐	☐	☐	☐
The following sessions were useful for me:					
Module 1: Two-ways thinking & impact of trauma	☐	☐	☐	☐	☐
Module 2: Physiology and neurobiology of trauma	☐	☐	☐	☐	☐
Module 3: Creating safe spaces	☐	☐	☐	☐	☐
Module 4: Building trusting respectful relationships	☐	☐	☐	☐	☐
Module 5: Beyond trauma informed care	☐	☐	☐	☐	☐

Did you learn anything new?

What were the things you liked most?

HPNF TRAINING EVALUATION

Were there parts we could have spent more time on?	
Were there parts you think we could have spent less time on?	

How did you hear about this training?

- [ ] Saw the poster
- [ ] From colleagues
- [ ] Staff newsletter
- [ ] Other, please describe
- [ ] My manager

Please add any additional comments

Thank you!

Supplementary File S5

Title of data: Knowledge. Attitudes, and Practice survey items.
Description of data: A table overview of the items included in the Knowledge, Attitudes, and Practice survey given to workshop participants at the beginning and end of each training session, and 3-months post-training

Item	Domain	Question
KAP1	Knowledge	There is evidence that trauma-informed approaches can improve outcomes for families.
KAP2	Attitude	Adopting a trauma-informed approach in my job is too hard.
KAP3	Attitude	Aboriginal and Torres Strait Islander families often find healthcare settings particularly distressing.
KAP4	Practice	I adjust how I say and do things to avoid retraumatising people.
KAP5	Attitude	Parents impacted by trauma are not able to provide nurturing care to their child/ren.
KAP6	Practice	I am able to create safe and welcoming spaces for people impacted by trauma.
KAP7	Knowledge	The colonisation of Australia has ongoing impacts for Aboriginal and Torres Strait Islander people today.
KAP8	Knowledge	To use trauma-informed approaches you must know specific details of a parent's trauma history.
KAP9	Practice	I take the time to build trusting relationships with families.
KAP10	Knowledge	I can name three local services for Aboriginal and Torres Strait Islander families.
KAP11	Knowledge	Closing the Gap targets 2 and 12 are on track to being achieved on time.
KAP12	Practice	I collaborate with internal and external service providers when working with Aboriginal and Torres Strait Islander families.
KAP13	Attitude	Racism is one of the reasons that more Aboriginal and Torres Strait Islander children are removed from their families into state care than non-Aboriginal children.

Additional items on three-month post-training survey

1. In the past three months have you applied any of the things you learned in the HPNF training?
2. Please describe the ways in which you have applied what you learned.
3. In the past three months, have you noticed any changes in the way care is provided at your service?
4. Please describe the changes you have noticed.

Supplementary File S6

Title of data: The TIDieR (Template for Intervention Description and Replication) Checklist
Description of data: The TIDieR (Template for Intervention Description and Replication) Checklist

**Item number**	**Item**	**Where located ****	
		Primary paper (page or appendix number)	Other † (details)
	**BRIEF NAME**		
**1**	Provide the name or a phrase that describes the intervention.	pp. 1, 5	
	**WHY**		
**2**	Describe any rationale, theory, or goal of the elements essential to the intervention.	pp. 6	
	**WHAT**		
**3**	Materials: Describe any physical or informational materials used in the intervention, including those provided to participants or used in intervention delivery or in training of intervention providers. Provide information on where the materials can be accessed (e.g. online appendix, URL).	pp. 6, 7, 8	
**4**	Procedures: Describe each of the procedures, activities, and/or processes used in the intervention, including any enabling or support activities.	pp. 7, 8	
	**WHO PROVIDED**		
**5**	For each category of intervention provider (e.g. psychologist, nursing assistant), describe their expertise, background and any specific training given.	pp. 8	
	**HOW**		
**6**	Describe the modes of delivery (e.g. face-to-face or by some other mechanism, such as internet or telephone) of the intervention and whether it was provided individually or in a group.	pp. 6, 8	
	**WHERE**		
**7**	Describe the type(s) of location(s) where the intervention occurred, including any necessary infrastructure or relevant features.	pp. 7	
	**WHEN and HOW MUCH**		

**8**	Describe the number of times the intervention was delivered and over what period of time including the number of sessions, their schedule, and their duration, intensity or dose.	pp. 7	
	**TAILORING**		
**9**	If the intervention was planned to be personalised, titrated or adapted, then describe what, why, when, and how.	NA	
	**MODIFICATIONS**		
**10‡**	If the intervention was modified during the course of the study, describe the changes (what, why, when, and how).	pp. 8, 24, 25	
	**HOW WELL**		
**11**	Planned: If intervention adherence or fidelity was assessed, describe how and by whom, and if any strategies were used to maintain or improve fidelity, describe them.	NA	
**12‡**	Actual: If intervention adherence or fidelity was assessed, describe the extent to which the intervention was delivered as planned.	NA	

** **Authors** - use N/A if an item is not applicable for the intervention being described. **Reviewers** – use '?' if information about the element is not reported/not sufficiently reported.

† If the information is not provided in the primary paper, give details of where this information is available. This may include locations such as a published protocol or other published papers (provide citation details) or a website (provide the URL).

‡ If completing the TIDieR checklist for a protocol, these items are not relevant to the protocol and cannot be described until the study is complete.

* We strongly recommend using this checklist in conjunction with the TIDieR guide (see *BMJ* 2014;348:g1687) which contains an explanation and elaboration for each item.

* The focus of TIDieR is on reporting details of the intervention elements (and where relevant, comparison elements) of a study. Other elements and methodological features of studies are covered by other reporting statements and checklists and have not been duplicated as part of the TIDieR checklist. When a **randomised trial** is being reported, the TIDieR checklist should be used in conjunction with the CONSORT statement (see www.consort-statement.org) as an extension of **Item 5 of the CONSORT 2010 Statement**. When a **clinical trial protocol** is being reported, the TIDieR checklist should be used in conjunction with the SPIRIT statement as an extension of **Item 11 of the SPIRIT 2013 Statement** (see www.spirit-statement.org). For alternate study designs, TIDieR can be used in conjunction with the appropriate checklist for that study design (see www.equator-network.org).

References

1. Chamberlain, C.; Gee, G.; Brown, S.J.; Atkinson, J.; Herrman, H.; Gartland, D.; Glover, K.; Clark, Y.; Campbell, S.; Mensah, F.K. Healing the Past by Nurturing the Future—Co-designing perinatal strategies for Aboriginal and Torres Strait Islander parents experiencing complex trauma: Framework and protocol for a community-based participatory action research study. *BMJ Open* **2019**, *9*, e028397. https://doi.org/10.1136/bmjopen-2018-028397.

2. Substance Abuse and Mental Health Services Administration. *SAMHSA's Concept of Trauma and Guidance for a Trauma-Informed Approach*. HHS Publication No. (SMA) 14-4884. Substance Abuse and Mental Health Services Administration: Rockville, MD, USA, 2014
